# CoO/Co_3_O_4_ heterostructures with intimate contact promote photocatalytic CO_2_ reduction

**DOI:** 10.1039/d6ra02788a

**Published:** 2026-04-30

**Authors:** Zhidong Yang, Xuelin Yang, Xikang Ding, Peixia Li

**Affiliations:** a School of Chemistry and Environment, Ankang University Ankang 725000 Shanxi China yangzd1618@163.com l485276@163.com

## Abstract

Here we delicately design CoO/Co_3_O_4_ heterostructures *via* a one-step solvothermal process for photocatalytic CO_2_ reduction. The intimate-contact heterojunction, in synergy with oxygen vacancies, direct the spatial separation of photogenerated charges and provide active reaction platforms, thus endowing CoO/Co_3_O_4_ with enhanced CO_2_-to-CO conversion performance.

## Abstract 2

A one-step solvothermal method is developed to fabricate CoO/Co_3_O_4_ heterojunctions with abundant oxygen vacancies that exhibit excellent photocatalytic CO_2_–CO-reduction performance.

## Graphical abstract

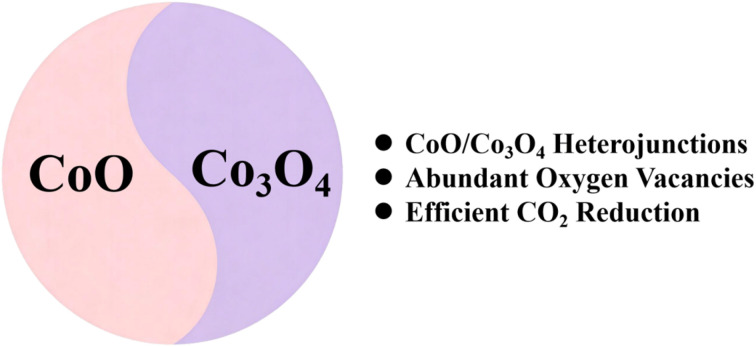

## 1 Introduction

Photocatalytic CO_2_ reduction represents a promising strategy that mimics natural photosynthesis to convert CO_2_ into valuable chemical fuels, offering an ideal solution to address global warming and energy shortage issues.^1–3^ The CO_2_ reduction reaction (CO_2_RR) involves a multi-electron process, wherein efficient extraction of photogenerated electrons represents a fundamental prerequisite for advanced photocatalysis.^4,5^ However, low availability of photogenerated electrons in intrinsic photocatalysts severely hinders their further application. Therefore, developing novel catalysts that are facile, cost-effective, and highly efficient in mediating the separation and migration of photogenerated charge carriers is critical to advancing high-performance photocatalytic CO_2_ reduction.^6^ Transition metal ions with diverse redox states serve as beneficial elements for establishing electron transport pathways,^7–9^ thereby enhancing multi-electron CO_2_ reduction. Co and Ni-based oxides (*e.g.*, Co_3_O_4_, CoO, NiO, NiCo_2_O_4_) are regarded as the most promising inexpensive yet highly efficient catalysts for the photochemical CO_2_RR, owing to their exceptional capability in enhancing charge transfer kinetics and interfacial reactions.^10–12^

Co_3_O_4_ serves as a promising photocatalyst owing to its distinctive crystal structure and electronic properties.^13–15^ The coexistent Co^2+^/Co^3+^ redox couples in its lattice act as intrinsic electron transport chains, where the strong electron-accepting capability of Co^3+^ enables rapid electron extraction from photoactive components or the reaction medium. The dynamic interconversion between Co^2+^ and Co^3+^ promotes charge transfer within the bulk phase, thereby mitigating charge accumulation to some extent. Moreover, the favourable electronic conductivity of Co_3_O_4_ facilitates the migration of extracted electrons, reducing charge transport resistance and ensuring sufficient electron supply for the CO_2_RR. However, the band structure of a single Co_3_O_4_ lacks flexible regulation, the charge separation efficiency is limited by the interface charge transfer barrier, and the directional migration of surface electron is insufficient, making it difficult to meet the practical application requirements of the multi-step CO_2_ reduction reaction. CoO is characterized by suitable band positions.^12^ The energy-level offset between CoO and Co_3_O_4_ gives rise to a robust built-in electric field at their interface, which not only enhances the electron extraction capability of Co_3_O_4_ but also constructs a directional charge transport channel, promoting the efficient transfer of electrons.^16^ Furthermore, the abundant surface oxygen vacancies and Co^2+^ active sites of CoO can further optimize the charge distribution and utilization efficiency.^12,17,18^

Herein, through the development of a facile one-step solvothermal approach, we demonstrate the fabrication of CoO/Co_3_O_4_ heterostructure catalysts with abundant oxygen vacancies for efficient photocatalytic CO_2_ reduction. The obtained CoO/Co_3_O_4_ heterostructures synergistically combine the structural and functional advantages of both intrinsic electron transport chains in Co_3_O_4_ and robust built-in electric field at the heterointerface, which constructs a directional charge transport channel to promote efficient electron transfer and significantly enhance charge separation efficiency. In addition, the *in situ* formation enables strong coupling between oxygen vacancies and CoO/Co_3_O_4_ heterostructures, which synergistically promotes photogenerated charge separation and CO_2_ adsorption/activation. Benefiting from the unique one-step synthesized CoO/Co_3_O_4_ heterostructures with enriched oxygen vacancies, the catalyst exhibits superior CO_2_ photoreduction activity and selectivity compared to single-phase CoO, Co_3_O_4_, providing new insights into the design of efficient heterostructure photocatalysts for CO_2_ reduction.

## 2 Experimental

### 2.1. Chemicals

Cobalt acetate (Co(CH_3_COO)_2_, 98%), cobalt(ii)oxide (CoO), ethanol absolute (CH_3_CH_2_OH, ≥99.7%) and sodium sulfate (Na_2_SO_4_, ≥99.0%) were purchased from Sinopharm Chemical Reagent Co., Ltd (China) and directly used without further purification. Ultrapure water for all solutions was obtained from a laboratory ultrapure water system (IA-30XV, Yihan, China).

### 2.2. Materials synthesis

#### 2.2.1. Preparation of CoO/Co_3_O_4_ heterostructure

CoO/Co_3_O_4_ was obtained *via* a solvothermal method. The typical synthetic experiments were as follows: 0.300 g of Co(CH_3_COO)_2_ was dissolved in 80 mL of a mixed solvent consisting of absolute ethanol and ultrapure water 9 : 1 (v/v) under ultrasonic assistance, yielding a transparent solution. The resulting solution was transferred into a 100 mL stainless steel autoclave equipped with a Teflon liner, which was subsequently heated in an oven at 180 °C for 12 h. After reaction, the product was dialyzed against ultrapure water for 48 h (molecular weight cutoff = 3500 Da) to remove residual impurities, followed by centrifugation to collect the solid product. The obtained product was washed with absolute ethanol several times and finally vacuum-dried at 60 °C.

#### 2.2.2. Preparation of T-Co_3_O_4_

2 g cobalt acetate was put into a ceramic boat and calcined at the temperature 673 K (5 K per min heating rate) for 4 h in air. After cooling to room temperature, the obtained catalyst was denoted as T-Co_3_O_4_.

### 2.3. Photocatalytic CO_2_ reduction tests

The photocatalytic CO_2_ reduction experiment was conducted on a MC-SPB10. Typically, 20 mg of photocatalysts was dispersed in 2 mL of deionized water under ultrasonication for 30 min, the suspension was dripped onto a rounded fiberglass paper with a diameter of 4 cm and naturally dried at ambient temperature. The sample-containing fiberglass paper was fixed to a tripod and placed in a Pyrex reaction vessel with 2 mL of deionized water. The reaction vessel was vacuumed three times and then pumped with high-purity CO_2_ (99.999%) to reach around 80 kPa. The temperature of whole reaction system was maintained at 25 °C by circulating condensed water. A 300 W Xenon lamp (MC-PF300C) was used to provide light source. The gas product was collected at an interval of 1 h and analyzed with an online gas chromatograph-GC9790II (PLF-01) equipped with flame ionization detector (FID) and thermal conductivity detector (TCD). For cyclic testing, specifically, after each photoreduction cycle, light irradiation was terminated. The catalyst was then carefully retrieved from the reactor and purged with high-purity nitrogen (Ar) for a certain duration to eliminate residual gases including CO_2_, H_2_O, and reaction products. Following this step, the catalyst was directly employed for the subsequent cycle without any additional processing.

### 2.4. Characterization techniques

X-ray diffraction (XRD) was performed on Bruker D8 Advance diffractometer. Morphology was performed using a Hitachi Regulus 8100 field-emission scanning electron microscope (FE-SEM). Transmission electron microscopy (TEM) and high-resolution TEM (HR-TEM) images were captured by a JEOL JEM-F200 field-emission transmission electron microscope. Electron paramagnetic resonance (EPR) spectra were collected at room temperature on a Bruker ELEXSYS-II E500 spectrometer. X-ray photoelectron spectroscopy (XPS) measurements were conducted on a Thermo Scientific K-Alpha instrument, with binding energies calibrated to the C 1s peak at 284.8 eV. Photoluminescence (PL) emission spectra were observed on a Fluorescence Spectrophotometer (Edinburgh FLS1000) with an excitation wavelength of 365 nm. CO_2_ temperature programmed desorption (TPD) measurements were conducted on the Micromeritics Autochem III2930 instrument. In a typical experiment, 100 mg of sample was first pretreated with helium gas (50 mL min^−1^) at 300 °C for 30 min and cooled down to 50 °C, and flowed with 10% CO_2_/He mixed gas for 30 min. After that, it was purged with helium gas for 1 h at 50 °C to remove weakly adsorbed CO_2_. Then the temperature was ramped to 600 °C at 10 °C min^−1^ under the flow of helium gas (30 mL min^−1^).

### 2.5. Photoelectrochemistry measurement

The electrochemical measurements were performed on an electrochemical workstation (CHI760C, Chinstruments, China) using a three-electrode system. The Pt sheet and Ag/AgCl electrode were used as the counter electrode and reference electrode, respectively. The electrolyte was Na_2_SO_4_ solution (0.2 M). The working electrode was prepared by dip-coating catalyst slurry on FTO conductive glass (1 × 1 cm) and then dried in the air.

## 3 Results and discussion

**Fig. 1:**
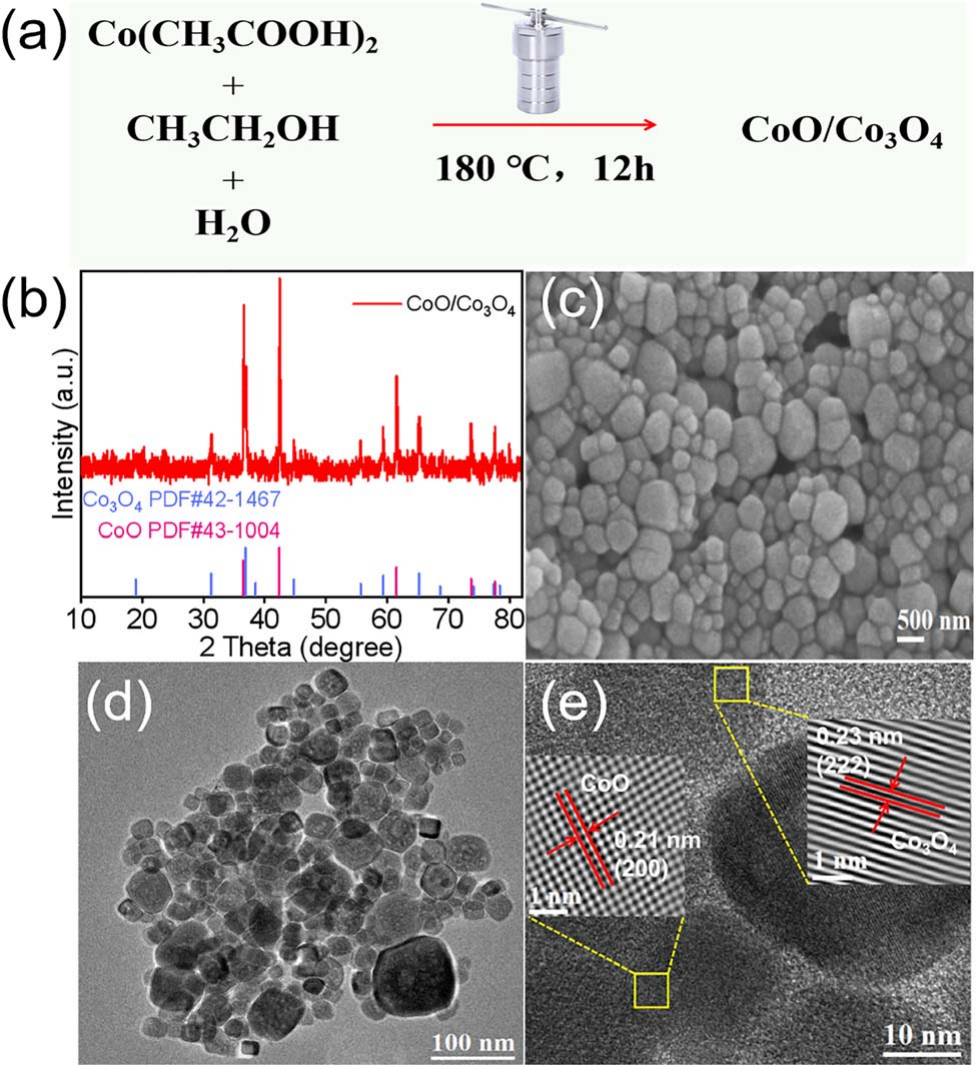
(a) Schematic illustration of the synthetic process of CoO/Co_3_O_4_. (b) XRD pattern of synthesized CoO/Co_3_O_4_. (c) FESEM and (d) TEM images of CoO/Co_3_O_4_. (e) HRTEM image of CoO/Co_3_O_4_.

The overall synthetic strategy involves a single solvothermal process, as schematically illustrated in Fig. 1a. During the process, the cobalt precursor in the ethanol–water mixed solvent undergoes hydrolysis and partial oxidation, leading to the partial conversion of Co^2+^ to Co^3+^. Subsequently, synchronous formation of CoO and Co_3_O_4_ phases occurs through phase separation crystallization, which further *in situ* self-assembles during the growth process to form a CoO/Co_3_O_4_ heterostructure with intimate interfacial contact. The crystal structure and phase composition of the synthesized materials are characterized using X-ray diffraction (XRD) analysis. As illustrated in Fig. 1b, the XRD pattern confirms the coexistence of cubic CoO (JCPDS no. 43-1004) and spinel Co_3_O_4_ (JCPDS no. 42-1467), thereby indicating the CoO/Co_3_O_4_ heterostructures has been successfully constructed. FT-IR spectroscopy measurements further corroborate the composite structure (Fig. S1). Two prominent absorption bands centered at ∼590 cm^−1^ and ∼658 cm^−1^ are attributed to the stretching vibrations of Co–O bonds in octahedral (Co^3+^) and tetrahedral (Co^2+^) coordination environments of spinel Co_3_O_4_, respectively.^19^ Meanwhile, a broad band centered at around 550–600 cm^−1^ corresponds to the characteristic Co–O stretching vibration of cubic CoO.^19,20^ Scanning electron microscope (SEM) and transmission electron microscope (TEM) characterization are used to explore the morphology of the catalyst samples. SEM image of the as-obtained CoO/Co_3_O_4_ reveals an irregular cubic morphology (Fig. 1c), which is further verified by TEM characterization (Fig. 1d). The high-resolution TEM (HRTEM) is also performed to explore the formation of CoO/Co_3_O_4_ at the atomic scale. As shown in Fig. 1e, the HRTEM image clearly reveals lattice fringes of 0.23 nm and 0.21 nm, corresponding to the (222) plane of Co_3_O_4_ and the (200) plane of CoO, respectively. An intimate contact is formed in CoO/Co_3_O_4_, which is critical for charge carrier separation and catalytic activity. The above results confirm the successful synthesis of the CoO/Co_3_O_4_ heterojunction composite *via* a one-step solvothermal method. For comparison, commercially available CoO (denoted as C-CoO) and Co_3_O_4_ derived from direct thermal decomposition (denoted as T-Co_3_O_4_) as references. The XRD and TEM images of the C-CoO and T-Co_3_O_4_ are shown in Fig. S2–5.

**Fig. 2:**
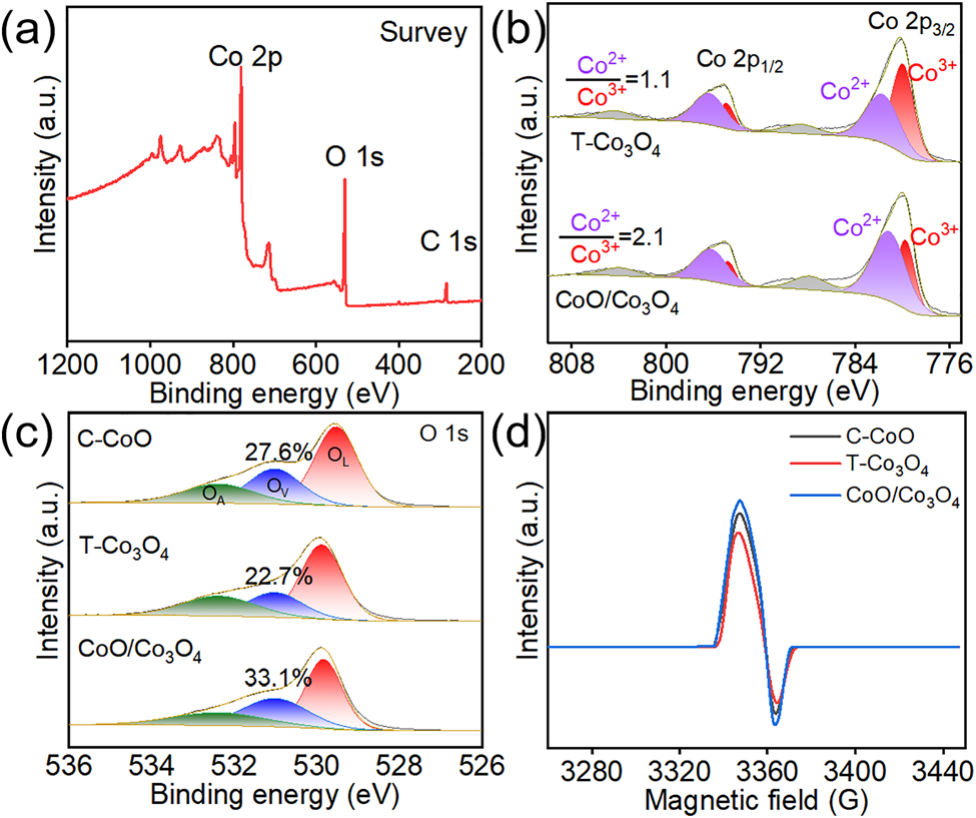
(a) XPS survey spectra of CoO/Co_3_O_4_. (b) High-resolution XPS spectra of Co 2p of T-Co_3_O_4_ and CoO/Co_3_O_4_. (c) High-resolution XPS spectra of O 1s of C-CoO, T-Co_3_O_4_ and CoO/Co_3_O_4_. (d) EPR spectra of C C-CoO, T-Co_3_O_4_ and CoO/Co_3_O_4_.

The chemical states and surface composition of the catalysts are analyzed using X-ray photoelectron spectroscopy (XPS). The survey XPS spectrum (Fig. 2a, S6 and S7) shows that the strong signals of C, Co and O element, confirming the concerned elements are present in the samples, in which the C element comes from the deposition of the atmosphere. The Co high-resolution XPS spectra of C-CoO, T-Co_3_O_4_ and CoO/Co_3_O_4_ are presented in Fig. 2b and S8. The binding energy values at around 780 ± 0.2 eV and 795 ± 0.2 eV correspond to Co 2p_3/2_ and Co 2p_1/2_, respectively. To investigate the electronic states of Co atoms, the high-resolution Co 2p spectra were fitted. The Co 2p_3/2_ and Co 2p_1/2_ peaks can be mainly fitted by two regions of Co^3+^ and Co^2+^. The peaks at 781.4 ± 0.2 and 796.2 ± 0.2 eV can be ascribed to Co^2+^, while the other two peaks at 779.7 ± 0.2 and 794.6 ± 0.2 eV correspond to Co^3+^. By comparing the areas of the fitted peaks, the Co^2+^ : Co^3+^ atomic ratio of CoO/Co_3_O_4_ is calculated to be 2.1, which is higher than that of T-Co_3_O_4_ (1.1). The high-resolution O 1s XPS spectrum shows three oxygen contributions (Fig. 2c), attributed to lattice oxygen (OL, 529.6 ± 0.2 eV), oxygen vacancy (OV, 531.2 ± 0.2 eV), and adsorbed water (OA, 532.4 ± 0.2 eV), respectively. Notably, the calculated ratio of 33.1% indicates that CoO/Co_3_O_4_ exhibits a high concentration of oxygen vacancies, arising from charge imbalance and lattice distortion at the heterogeneous interface. Electron paramagnetic resonance (EPR) spectra are also employed to analyse the formation of OVs, as shown in Fig. 2d. All samples display an EPR signal at *g* = 2.001, which can be attributed to the OVs signal.^21,22^ The EPR intensity increases with the increase of OVs concentration, validating the observation of XPS.

**Fig. 3:**
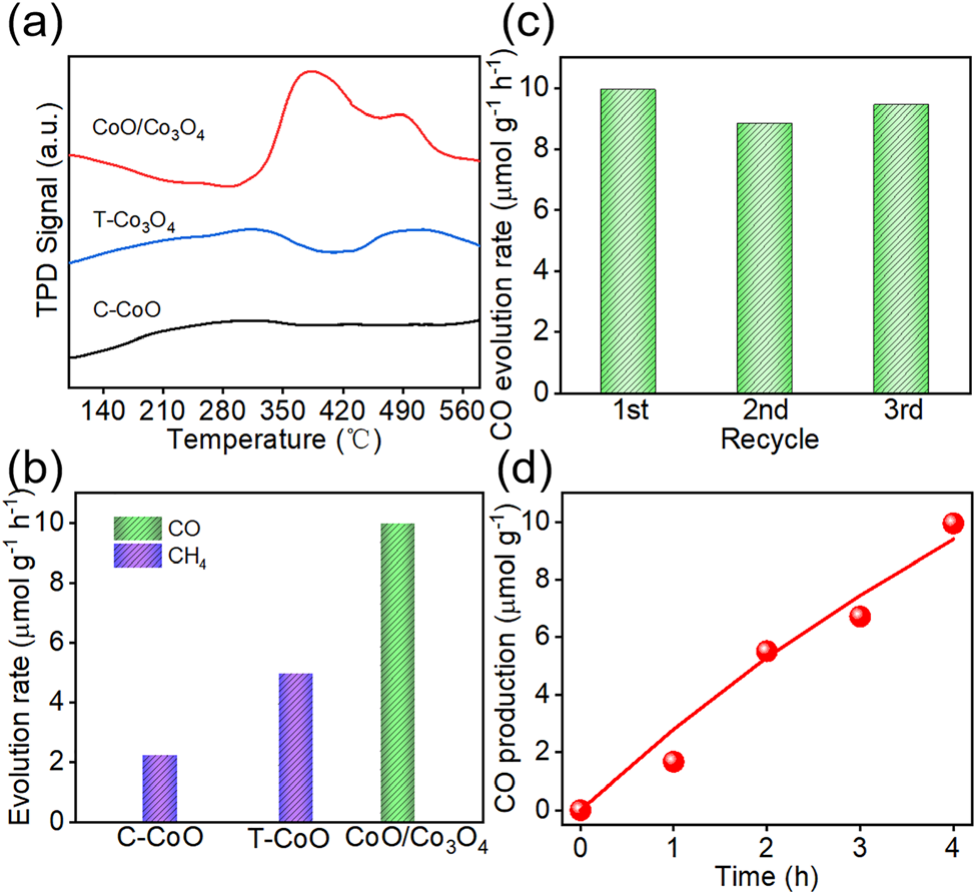
(a) CO_2_-TPD profiles and (b) photocatalytic CO_2_ reduction activities of C-CoO, T-Co_3_O_4_ and CoO/Co_3_O_4_. (c) Photocatalytic CO_2_ cycle stability of CoO/Co_3_O_4_. (d) Time courses of photocatalytic activities of CO over CoO/Co_3_O_4_.

The capacity of catalysts to capture and activate CO_2_ is a critical determinant of CO_2_ reduction efficiency. CO_2_ temperature-programmed desorption (CO_2_-TPD) was employed to probe the thermodynamic and kinetic aspects of CO_2_ adsorption. As shown in the TPD profiles (Fig. 3a), C-CoO exhibits a broad and weak desorption signal in the 200–350 °C range, attributed to intermediate-strength chemisorption on low-coordinated Co^2+^ cations and lattice oxygen. In contrast, T-Co_3_O_4_ displays two moderate peaks at 260–370 °C and at 430–560 °C, assigned to CO_2_ adsorption on highly acidic Co^3+^ sites and strong basic sites associated with oxygen vacancies, respectively.^23,24^ Strikingly, the CoO/Co_3_O_4_ photocatalyst features a reconfigured desorption profile with optimized shape and temperature distribution: it exhibits both a strong CO_2_ desorption peak in 320–430 °C and a distinct signal in 460–520 °C. Notably, stronger catalyst-CO_2_ interactions correlate with higher desorption temperatures, indicating enhanced binding strength. The improved CO_2_ adsorption capacity originates from abundant chemisorption sites generated by lattice mismatch and defects at the heterointerface. Collectively, heterojunction construction enables efficient CO_2_ adsorption-activation, largely stemming from interfacial electronic effects and enriched oxygen vacancies.

The catalytic performances of all samples are evaluated by a 300 W Xe arc lamp light driven CO_2_ photoreduction reactions in the gas phase, without the use of any cocatalyst and sacrificial agent. As shown in Fig. 3b, methane (CH_4_) and carbon monoxide (CO) are detected as the products from CO_2_ reduction. Due to its relatively weak CO_2_ adsorption capacity, C-CoO exhibits a rather low photocatalytic activity, with a total CO_2_ conversion rate of 2.21 µmol g^−1^ h^−1^. T-Co_3_O_4_ shows a much better CO_2_ reduction performance (total CO_2_ conversion 4.95 µmol g^−1^ h^−1^). After the construction of the heterojunction, the CO_2_ conversion rate for CoO/Co_3_O_4_ photocatalyst is greatly enhanced to 9.95 µmol g^−1^ h^−1^, which is about 5 and 2 times higher than the corresponding C-CoO and T-Co_3_O_4_ values respectively. The role of oxygen vacancies and heterostructure in the CoO/Co_3_O_4_ heterostructure may further amplify CO selectivity by modulating intermediate adsorption and charge separation.^25–29^ The achieved CO_2_ reduction rate is superior to that of many other systems (Table S1, SI). The time courses for the yields of CO are depicted in Fig. 3c. The stability of the CoO/Co_3_O_4_ catalyst is then evaluated. No obvious performance decrease is observed during the three-cycle test (Fig. 3d), suggesting preliminary stability of CoO/Co_3_O_4_ heterostructures, along with designating long-term durability evaluation as a key direction for future work. These results indicate that this heterojunction catalytic system is mainly responsible for the CO_2_-to-CO transformation reaction with considerable efficiency. The CoO/Co_3_O_4_ heterostructure is capable of enhancing the adsorption/activation of CO_2_ molecules and accelerating the transport kinetics of photogenerated charges. No hydrocarbon products are detected from the developed system, consistent with the results of reported works.

**Fig. 4:**
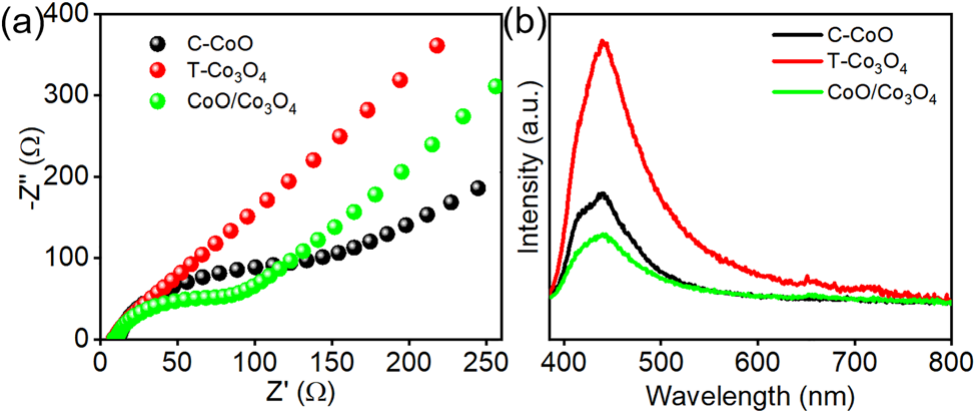
(a) EIS Nyquist plots and (b) PL spectra of C-CoO, T-Co_3_O_4_ and CoO/Co_3_O_4_.

Photoelectrochemical characterizations were performed to unravel the critical role of the CoO/Co_3_O_4_ heterostructure in promoting charge carrier transfer kinetics during CO_2_ photoreduction. The EIS Nyquist plot in Fig. 4a exhibits two distinct semicircles: the first semicircle corresponds to the high-frequency region, where the CoO/Co_3_O_4_ heterostructure shows significantly lower resistance compared to both individual C-CoO and T-Co_3_O_4_ components. This critical range directly relates to charge transfer efficiency, which is essential for CO_2_ photoreduction. The enhanced charge transfer may be attributed to the synergistic effect of the heterostructure and enriched oxygen vacancies, facilitating efficient carrier separation and transfer. To further probe the separation and recombination dynamics of photogenerated charge carriers and the origin of improved photocatalytic performance, photoluminescence (PL) measurements *λ*_exc_ = 365 nm are conducted (Fig. 4b). Notably, the CoO/Co_3_O_4_ catalyst shows a remarkably quenched PL intensity. The PL quenching principally reflects an inhibited recombination rate of charge carriers,^30^ thereby substantially promoting heterogeneous CO_2_ conversion. Fig. S9 shows the transient photocurrent curves of the samples during four on–off cycles. The current density of the CoO/Co_3_O_4_ is significantly higher than that of the C-CoO and T-Co_3_O_4_, indicating an enhanced separation rate of electron–hole pairs, agreeing with the results of semicircular Nyquist plots, PL. These photoelectrochemical observations demonstrate that the CoO/Co_3_O_4_ heterostructure effectively enhances the separation and transport of photogenerated charge carriers in the CO_2_ reduction system, leading to a significant improvement in catalytic performance.

## 4 Conclusions

In summary, CoO/Co_3_O_4_ heterostructures are rationally constructed as a highly efficient photocatalyst for CO_2_ reduction. These heterointerfaces hold unique electronic and interfacial features with an intimate contact for drastically enhanced separation, optimized charge carrier dynamics, as well as improved CO_2_ adsorption and activation. Consequently, the CoO/Co_3_O_4_ catalyst exhibits remarkable photocatalytic performance for the CO_2_-to-CO conversion rate. All these findings demonstrate that the rational design and construction of heterostructures can be an efficient strategy to achieve highly active earth-abundant materials for CO_2_ photoreduction. This work might encourage the study on heterointerfacial engineering for advanced artificial photosynthetic systems in solar energy-related applications.

## Author contributions

Zhidong Yang: conceptualization, funding acquisition, supervision, writing – review & editing. Peixia Li: project administration, methodology, writing – original draft. Xuelin Yang: investigation, visualization. Xikang Ding: validation.

## Conflicts of interest

There are no conflicts to declare.

## Supplementary Material

RA-016-D6RA02788A-s001

## Data Availability

The data supporting this article have been included as part of the supplementary information (SI). Supplementary information is available. See DOI: https://doi.org/10.1039/d6ra02788a.
