# Supplementary material for: CoO/Co3O4 heterostructures with intimate contact promote photocatalytic CO2 reduction
Source: RSC Adv. 2026 Apr 30;16(25):22559–63. doi: 10.1039/d6ra02788a (PMC13129719; doi:10.1039/d6ra02788a)
Supplement: RA-016-D6RA02788A-s001 [file RA-016-D6RA02788A-s001.pdf]

Supplementary Information (SI) for RSC Advances.
This journal is © The Royal Society of Chemistry 2026

Supporting Information

CoO/Co_3O_4 heterostructures with intimate contact promote photocatalytic CO_2 reduction

Zhidong Yang,* Xuelin Yang, Xikang Ding and Peixia Li *

School of chemistry and Environment, Ankang University, Ankang 725000, Shanxi, China.

*Corresponding authors:

E-mail: yangzd1618@163.com; l485276@163.com.

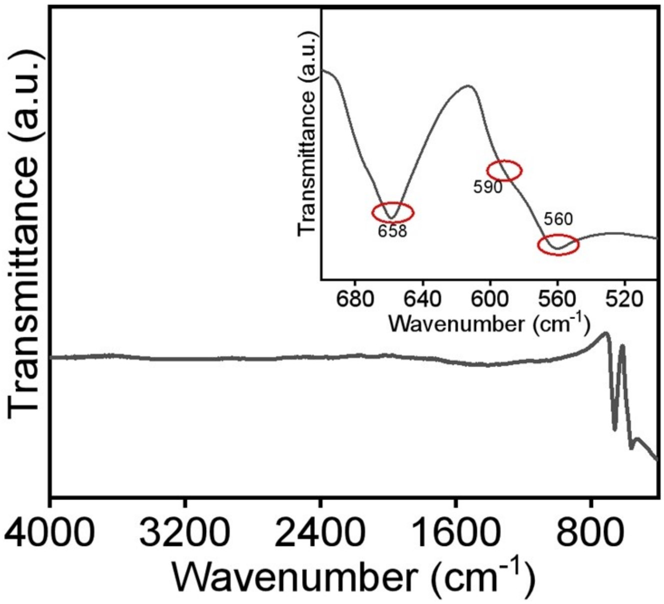

Fig. S1 FT-IR spectrum of obtained CoO/Co_3O_4.

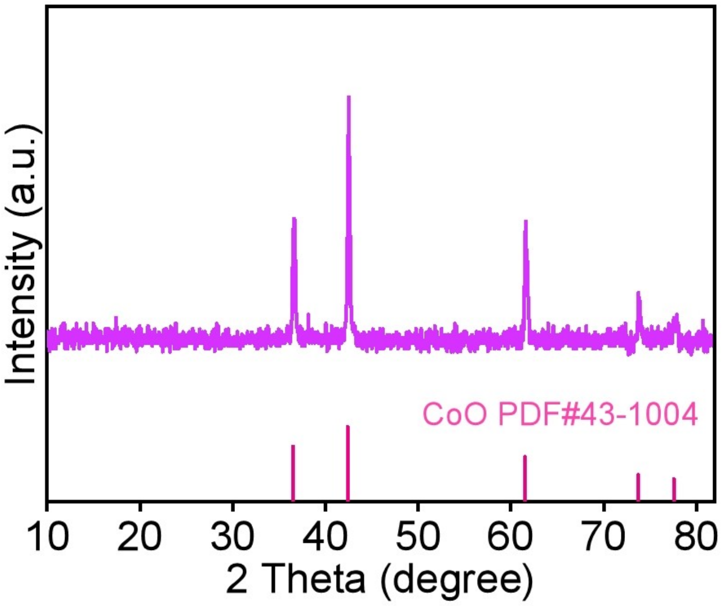

Fig. S2 X-ray diffraction (XRD) pattern of C-CoO.

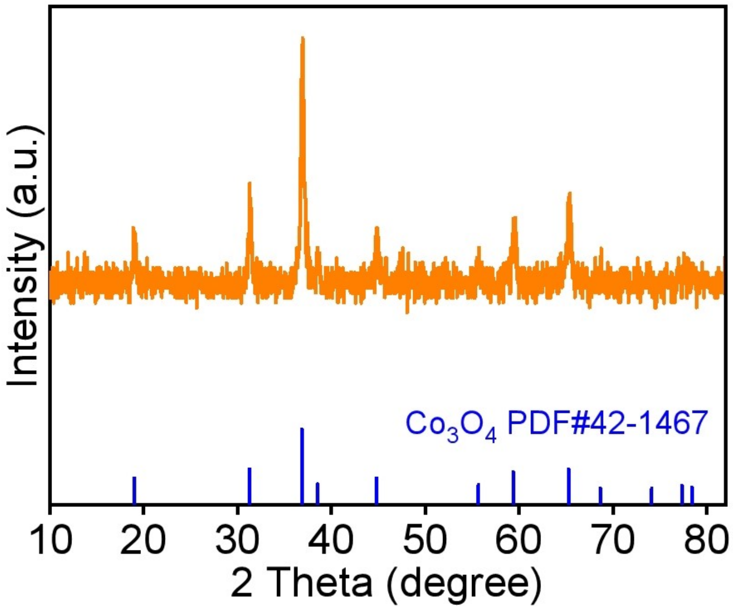

Fig. S3 X-ray diffraction (XRD) pattern of T-Co_3O_4.

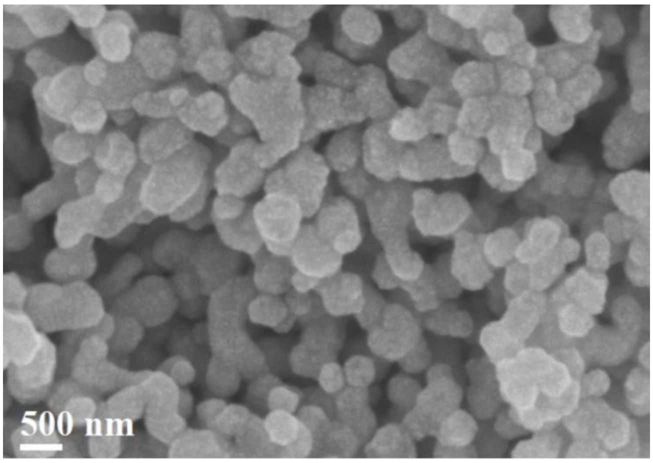

Fig. S4 Field emission scanning electron microscopic (FESEM) image of C-CoO.

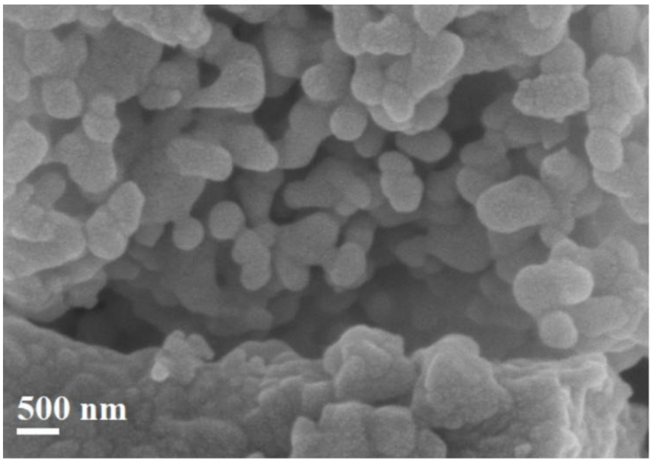

Fig. S5 Field emission scanning electron microscopic (FESEM) image of T-Co_3O_4.

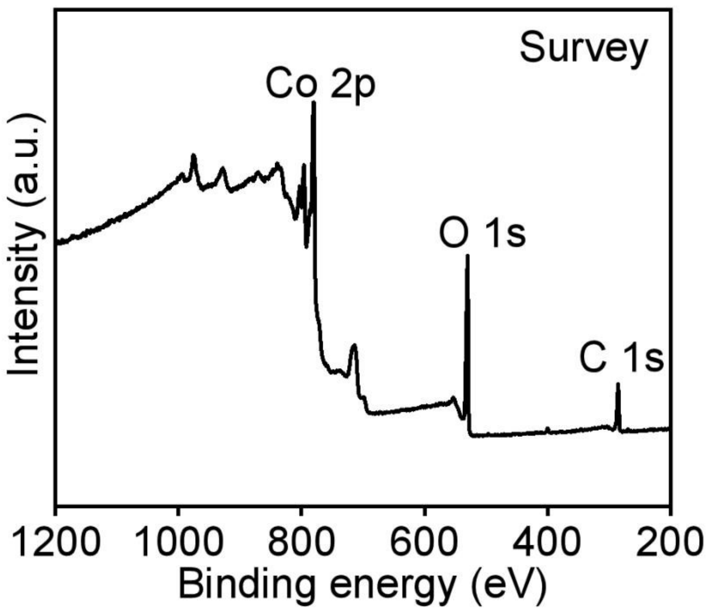

Fig. S6 XPS survey spectra of C-CoO.

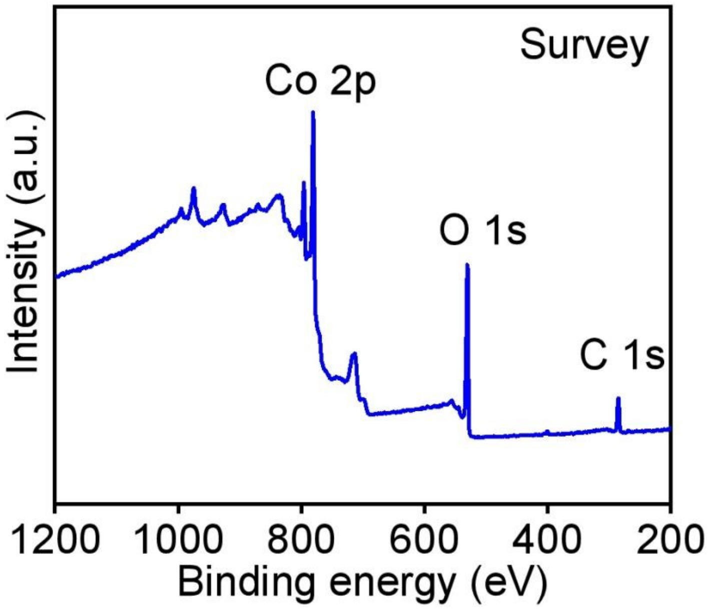

Fig. S7 XPS survey spectra of T-Co_3O_4.

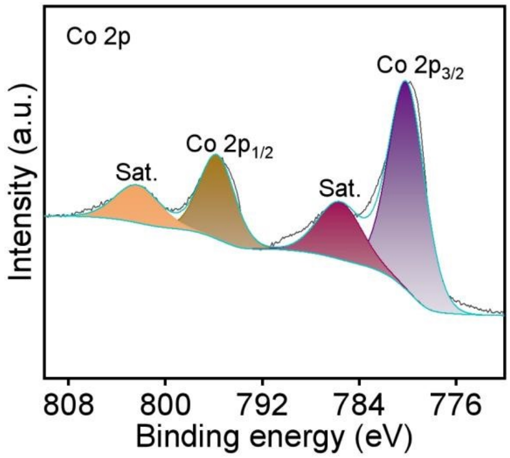

Fig. S8 High-resolution XPS spectra of Co 2p of C-CoO.

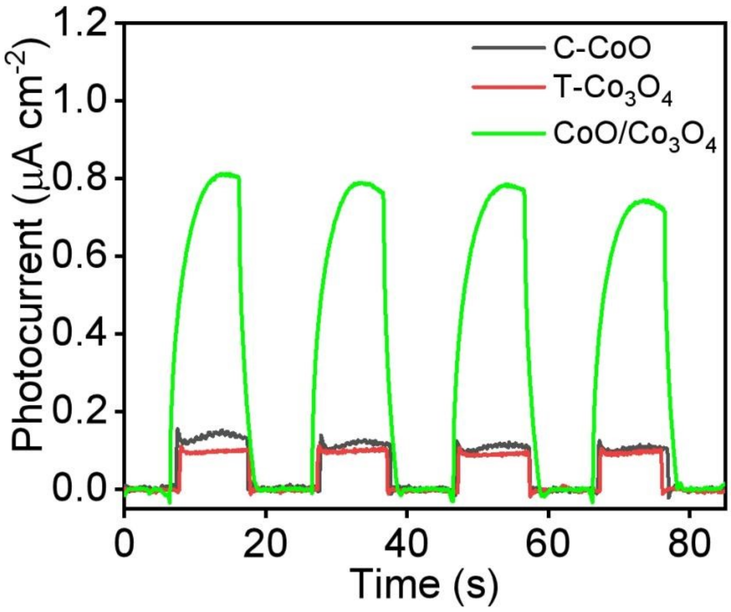

Fig. S9 Transient photocurrent curves of C-CoO, T-Co_3O_4 and CoO/Co_3O_4.

Table S1. Comparison of the photocatalytic CO_2 reduction activities of metal oxide-based photocatalysts.

Photocatalyst	Light source	Conditions	Dosage	CO (μmol g^{-1} h^{-1})	CH_4 (μmol g^{-1} h^{-1})	Ref
CoO@N-GC-500	UV-Vis light: 300 W Xe lamp	CO_2 and H_2O vapor	50 mg	5.16	10.03	1
Co@Ni/GC-700	UV-Vis light: 300 W Xe lamp	CO_2 and H_2O vapor	50 mg	4.89	9.79	2
ZnO@Co_3O_4	UV-Vis light: 300 W Xe lamp	CO_2 and H_2O vapor	100 mg	6.50	0.99	3
NiO/g-C_3N_4	UV-Vis light: 300 W Xe lamp	CO_2 , H_2O, triethanolamine	20 mg	2.75	1.79	4
MnO_2/g-C_3N_4	UV-Vis light: 300 W Xe lamp	CO_2 and H_2O vapor	50 mg	/	9.6	5
15%CuO/g-C_3N_4	UV-Vis light: 300 W Xe lamp	CO_2 and H_2O vapor	50 mg	3.78	/	6
Ordered mesoporous TiO_2	UV-Vis light: 300 W Xe lamp	CO_2 and H_2O vapor	100 mg	0.19	0.15	7
Co-OMT-4	UV-Vis light: 300 W Xe lamp	CO_2 and H_2O vapor	100 mg	2.09	0.33	8
CoO/Co_3O_4	300W Xe lamp		20 mg	9.95	/	This work

References

1 L. He, W. Zhang, S. Liu, Y. Zhao, Appl. Catal. B-Envrion., 2021, 298, 120546.

2 L. He, W. Zhang, S. Liu, Y. Zhao, J. Alloy Compd.,2023, 934, 168053.

3 T. Wang, L. Shi, J. Tang, V. Malgras, S. Asahin, G. Liu, H. Zhang, X. Meng, K. Chang, J. He, O. Terasaki, Y. Yamauchi, J. Ye, Nanoscale, 2016, 8, 6712-6720.

4 L. Wang, Y. Dong, J. Zhang, F. Tao, J. Xu, J. Solid State Chem., 2022, 308, 122878.

5 M. Wang, M. Shen, L. Zhang, J. Tian, X. Jin, Y. Zhou, J. Shi, Carbon, 2017, 120, 23-31.

6 M. Li, Y. Wu, E. Gu, W. Song, D. Zeng, J. Alloy Compd., 2022, 914, 165339.

7 T. Wang, X. Meng, P. Li, S. Ouyang, K. Chang, G. Liu, Z, Mei, J. Ye, Nano Energy, 2014, 9, 50-60.

8 T. Wang, X. Meng, G. Liu, K. Chang, P. Li, Q. Kang, L. Liu, M. Li, S. Ouyangcd, J. Ye, J. Mater. Chem. A, 2015, 3, 9491-9501.
